# Dynamical Transitions and Diffusion Mechanism in DODAB Bilayer

**DOI:** 10.1038/s41598-018-19899-6

**Published:** 2018-01-30

**Authors:** P. S. Dubey, H. Srinivasan, V. K. Sharma, S. Mitra, V. Garcia Sakai, R. Mukhopadhyay

**Affiliations:** 1 Solid State Physics Division, Bhabha Atomic Research Centre, Mumbai, 400085 India; 2 ISIS Facility, Science and Technology Facilities Council, Rutherford Appleton Laboratory, Didcot, UK; 3 Homi Bhabha National Institute, Anushaktinagar, Mumbai, 400094 India

## Abstract

Dioctadecyldimethylammonium bromide (DODAB), a potential candidate for applications in drug transport or DNA transfection, forms bilayer in aqueous media exhibiting a rich phase behavior. Here, we report the detailed dynamical features of DODAB bilayer in their different phases (coagel, gel and fluid) as studied by neutron scattering techniques. Elastic intensity scans show dynamical transitions at 327 K in the heating and at 311 K and 299 K during cooling cycle. These results are consistent with calorimetric studies, identified as coagel-fluid phase transition during heating, and fluid-gel and gel-coagel phase transitions during cooling. Quasielastic Neutron Scattering (QENS) data analysis showed presence of only localized internal motion in the coagel phase. However, in the gel and fluid phases, two distinct motions appear, namely lateral motion of the DODAB monomers and a faster localized internal motion of the monomers. The lateral motion of the DODAB molecule is described by a continuous diffusion model and is found to be about an order of magnitude slower in the gel phase than in the fluid phase. To gain molecular insights, molecular dynamics simulations of DODAB bilayer have also been carried out and the results are found to be in agreement with the experiment.

## Introduction

Lipids and surfactants are amphiphilic molecules, comprised of a hydrophilic head and hydrophobic hydrocarbon chain(s) that self-assemble under favorable conditions in aqueous medium, to form micelles, vesicles, liposomes or even more complex structures. Liposomes of synthetic lipids serve as a model system to study the more complex cell membrane that is mainly composed of lipids and proteins^1–5^. There is a growing interest in studying liposomes for their various biomedical applications in drug/vaccine delivery^6–8^, gene/DNA transfection^9,10^, or antimicrobial/antifungal action^11^, to name a few.

The first synthetic bilayer^12^ structure was formed from Dioctadecyldimethylammonium bromide (DODAB), a simple organic compound that has been the subject of several studies in the recent years^13–29^. Owing to the ability of DODAB to form stable bilayer vesicles with membrane-mimicking properties, the morphological changes of DODAB bilayers are considered as a model system to understand biological processes such as endocytosis^5,13^. Moreover, the DODAB liposome and its complexes are of great interest to study stability and solubilization of liposome-drugs and liposome-gene/DNA complexes^14–18^. In a very recent *in-vivo* study, DODAB-CeO_2_ hybrid nanoparticles were found to be efficient nonviral vectors for gene delivery in mammalian cells^19^. Sonication of DODAB aqueous dispersions leads to the formation of DODAB bilayer fragments (BF), which are an excellent option for hydrophobic drug and vaccine encapsulation^6–8^. Being a quaternary ammonium compound, DODAB by itself is also useful as antimicrobial and antifungal agent^11^. In the view of these biophysical properties with possible high-impact applications, it is paramount to understand the various aspects of the phase behavior of DODAB dispersions.

**Figure 1:**
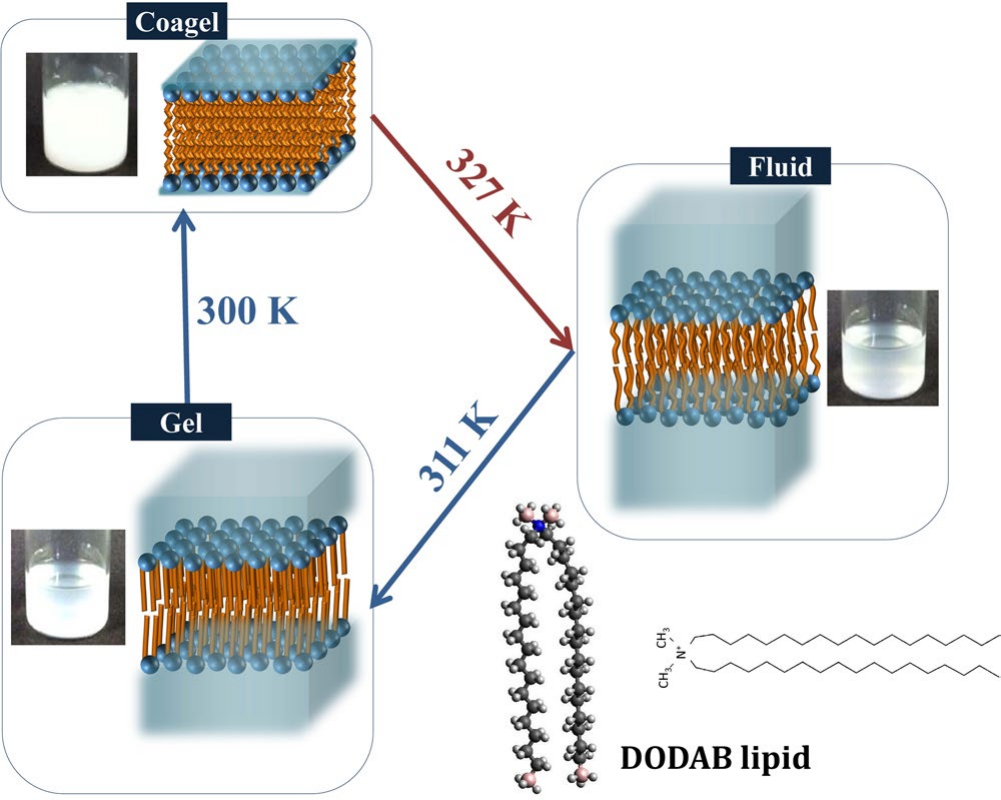
Photographs of a DODAB dispersion (70 mM) in the different phases along with the schematic of molecular ordering in the bilayer. The water content in interlamellar spaces decreases progressively as one goes from fluid to gel and from gel to coagel phases.

Over the last decade, a variety of techniques have been employed to unravel the complex phase diagram of DODAB bilayers^20–26^, which can now be traced out with four major phases – coagel, subgel, gel and fluid/liquid crystalline^24–26^. Below the chain melting temperature (T_m_), hydrated DODAB bilayer can be in gel, subgel or coagel phases depending on the concentration and temperature. In all these phases, the lipid chains are ordered, nearly in all*-trans* configuration, but differ in their packing density, hydration and dynamics. While the alkyl chain packing in the gel phase is hexagonal, it transforms to a much more tightly packed triclinic structure in the subgel and coagel phases^24^. Although the chain packing between the subgel and coagel phases is not much different, Fourier Transform Infrared (FTIR) measurements suggest that the headgroups in the coagel phase are highly dehydrated compared to the subgel and gel phases^24,26^. In fact, microscopy and SAXS measurements indicate that the coagel phase has a crystalline multilamellar packing with hardly any interlamellar water (Fig. 1) whereas the subgel is found to be made of large unilamellar faceted vesicles^24–26^. Upon melting any of the three gel phases, the fluid phase appears where lipid molecules are disordered. It is observed that the T_m_ depends on the sample preparation method and on the counterion nature^22^. The formation of subgel or coagel phases below T_m_ depends on the concentration of the system. Only beyond sufficiently high concentrations (>65 mM) the formation of coagel is observed^23,24^. In the low concentration regime, upon heating, the subgel phase transforms into the fluid phase via an intermediate gel phase. However, in the high concentration regime (>65 mM), the coagel phase directly melts into the fluid phase^23^. But interestingly, the intermediate gel phase is observed in the cooling cycle even in the high concentration regime^24,26^. It is suggested that this intermediate gel phase in the cooling cycle is due to the nonsynchronous change in the head and tail group of the DODAB bilayer during cooling^26^. Actual photographs of DODAB dispersion (70 mM) in the different phases along with the schematic of molecular ordering of the bilayer are shown in Fig. 1.

The dynamics of lipids in a membrane is important to understand various processes like cell-signaling, membrane-protein interaction, permittivity, endocytosis, etc. The lipid dynamics also plays a decisive role in drug encapsulation and transport by vesicles^30,31^. Therefore, a detailed knowledge of lipid dynamics within the membrane leaflets is necessary for a deep understanding of transport properties and of great relevance for pharmaceutical applications. Moreover, together with structural and morphological properties of DODAB, the molecular dynamics of lipids in different phases can provide us with a more comprehensive picture of the system’s phase behavior. Quasielastic neutron scattering (QENS) is a suitable technique to investigate molecular motions at the length scale of few angstroms and timescales of a few picoseconds to several nanoseconds^32^. It has been extensively used to probe molecular mobility in lipid/surfactant self-assemblies^30,33–48^. QENS provides information of both the geometry and timescale of the molecular motions in a system, although it can only probe the average atomic behavior of a macroscopic sample. Complementarily, molecular dynamics (MD) simulations provides insights about dynamical processes with atomistic resolution, and can overcome the experimental limitations due to an averaged system response, thus helping to pinpoint the role played by specific atomic groups. Given the computing resources at hand nowadays, MD simulations can probe molecular motions in spatial and temporal domains similar to QENS, and therefore give us the opportunity to directly validate a physical model used to describe the QENS data.

Here we report a detailed dynamical behavior of DODAB vesicles across its different phases, using neutron elastic intensity scans and QENS techniques, coupled with classical MD simulations and complemented by differential scanning calorimetry (DSC).

## Material and Methods

### Materials and Sample Preparation

DODAB, (C_18_H_37_)_2_N(CH_3_)_2_Br powder (>98%) and D_2_O (99.9% atom D purity) are obtained from Tokyo Chemical Industries Co. LTD. and Aldrich respectively. 70 mM DODAB vesicles were prepared by mixing the appropriate amounts of DODAB powder in D_2_O. The obtained mixture was kept under magnetic stirring at 65 °C for 30–45 min, which yielded an almost transparent fluid as shown in Fig. 1. The obtained DODAB dispersion was then equilibrated for a day.

### DSC Measurements

DSC measurements were carried out on the equilibrated DODAB solution using a Mettler Toledo DSC instrument. The DSC measurements were performed in the temperature range 285–345 K. The heating and cooling rate were maintained at 5 K/min.

### Neutron Scattering Measurements

Neutron scattering experiments were carried out on DODAB dispersions using IRIS, a backscattering spectrometer at the ISIS pulsed Neutron and Muon source at the Rutherford Appleton Laboratory, UK. IRIS is an indirect geometry neutron spectrometer, with a PG(002) analyzer that, in the offset mode, offers an energy resolution ΔE = 17 μeV (full width at half-maximum) and an accessible range of energy transfer from −0.3 to +1.0 meV. The available *Q*-range in the chosen set up was 0.5 to 1.8 Å^−1^. For neutron scattering measurements, the DODAB vesicles samples were placed in annular aluminum sample cans with 0.5 mm internal spacing such that the sample scattering was no more than 10%, thereby minimizing multiple scattering effects. Two types of measurements were carried out: (i) fixed elastic window scan (FEWS) as a function of temperature and (ii) Quasielastic Neutron Scattering (QENS) experiments. In FEWS measurements, the integrated elastic intensity within the energy resolution of the spectrometer is recorded as a function of temperature. FEWS experiments have been carried out during both heating and cooling cycles, in the temperature range 285–345 K, to look for the presence of dynamical transitions, their reversibility, as well as any hysteresis involved with them. In QENS measurements, the scattering function S(Q, E) is obtained, which provides information regarding the dynamical features of the system. Further details of the FEWS and QENS techniques are discussed in results and discussion. The measurements were carried out at 315 and 345 K during the heating cycle, and at 330, 315 and 308 K during the cooling cycle. The temperatures at which QENS data were recorded were based on the phase transitions observed in FEWS and DSC scans. QENS measurements on DODAB vesicles were also carried out at 10 K, which was used for normalizing the FEWS data for extracting the mean squared displacement (MSD). Instrument resolution was obtained by measuring the QENS data from standard vanadium sample. Further QENS data were recorded from pure D_2_O at 308, 315, 330 and 345 K for reference. MANTID software^49^ was used to perform standard data reduction.

### Molecular Dynamics Simulation Details

A bilayer of Dioctadecyldimethylammonium ion (DODA^+^) molecules was built using the Packmol software^50^ with 64 monomers in each monolayer, such that the tails of DODA^+^ ions were facing each other. The resultant bilayer was then solvated by adding 3600 water molecules and 128 bromide ions. The CHARMM-27 forcefield^51^ was used to parameterize DODA^+^ molecules using the DL_FIELD package^52^. The parameters for Bromide ion were obtained from a recent work by Joung and Cheatham^53^. The TIP3P model was used for water as an explicit solvent^54^. The whole system was simulated using DL_POLY-4^55^ using the Langevin barostat and thermostat to maintain a NPT ensemble. Long range interactions were treated using Particle mesh Ewald sum with a real space cut-off of 10 Å. MD simulations were carried out at 350 K, well above the transition temperature, to study specifically the fluid phase of the system. The system was simulated at 298 K, which in principle corresponds to an ordered phase of DODAB bilayer. Subsequent to an equilibration of 15 ns, a production run of 5 ns was carried out where the positions of the atoms in the system were recorded every 4 ps. A separate short run of 10 ps, with a recording frequency of 0.02 ps, was also carried out to capture trajectories of atoms at short times. The MD simulation runs of 20 ns were carried out with two different initial conditions to check the robustness of our simulations and the validity of the simulated results.

## Results and Discussion

**Figure 2:**
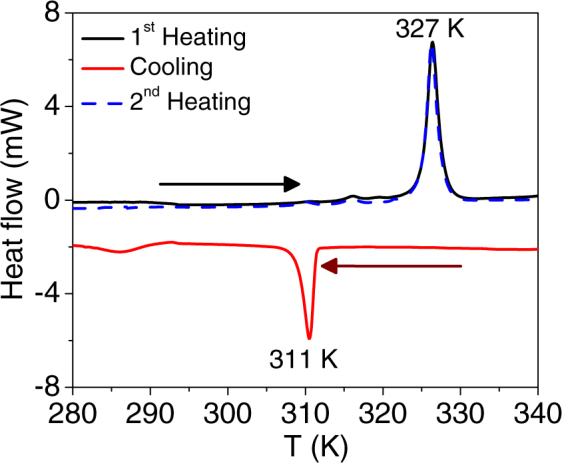
Differential scanning calorimetry (DSC) data from 70 mM DODAB bilayers. The sharp endothermic peak at 327 K during heating cycle corresponds to transition from coagel to fluid phase. In cooling cycle, a sharp exothermic peaks at 311 K and a broad hump at 284–292 K correspond to fluid to gel and gel to coagel phase transitions, respectively are observed. DSC thermogram in 2^nd^ heating cycle is also shown which is identical to 1^st^ heating cycle.

To investigate the phase behavior of DODAB bilayers, DSC measurements were carried out on 70 mM DODAB vesicles in heating and cooling cycles. In the heating cycle, a sharp endothermic peak is observed at 327 K; while in the cooling cycle, a sharp exothermic peak at 311 K and a broad exothermic hump at 284–292 K are observed as shown in Fig. 2. The observed DSC thermogram is found to be consistent with the reported results^23,24^ on the thermotropic phase behavior of DODAB. It has been shown^24^ that, in this concentration range, the coagel phase is the most stable one at low temperature and, upon heating, it transforms directly into the fluid phase. However, while cooling, the system first undergoes a fluid-to-gel transition and then a further transition from gel to coagel takes place at much lower temperature. It may be noted that in the coagel phase the head group region is highly dehydrated^26^, but the dehydration of head groups happens only after the rearrangement of lipid tails. This nonsynchronicity in transformation of head and tail regions of the DODAB lipid is the key issue to the formation of the gel phase^25^. The endothermic peak at 327 K in the heating cycle corresponds to the coagel-to-fluid phase transition, whereas in the cooling cycle the exothermic peak at 311 K and broad hump at 284–292 K correspond to the fluid to gel and gel to coagel transitions respectively. DSC measurements have also been carried out for a second heating cycle and the thermogram observed is the same as in the first heating cycle. This indicates that the state at the lowest temperature of the cooling cycle is actually the coagel phase^24^.

As mentioned earlier, FEWS – i.e. fixed elastic window scans as a function of temperature – is a suitable technique to observe the microscopic dynamical counterpart involved in phase transitions. A FEWS signal is produced by dynamical processes occurring at energies lower than the instrumental energy resolution (17 μeV in the present case), or in other words by atoms undergoing slow dynamics with characteristic times smaller than the inverse energy resolution. On the other hand, as the system temperature gets high enough to shift any dynamical process outside the elastic energy window of the spectrometer – i.e. atomic motions get faster than the instrumental time scale – a drop in elastic intensity is observed. Therefore, any abrupt change (loss in heating cycle and gain in cooling cycle) of elastic intensity in FEWS is a signature of a phase transition that is associated with a sudden change in the dynamics of the system.

**Figure 3:**
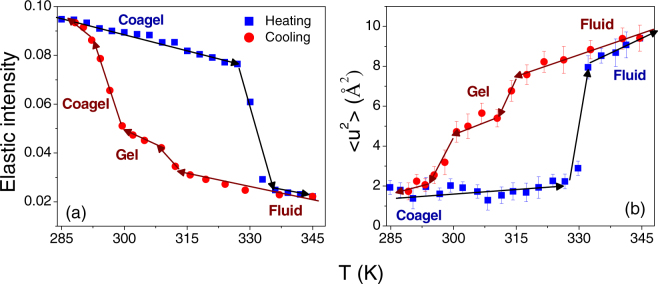
(**a**) *Q*-averaged (0.5 < Q(Å^−1^) <1.8) elastic intensity as observed for 70 mM DODAB vesicles in the temperature range of 285–345 K. In heating cycle, a sharp fall in elastic intensity at 327 K corresponds to the coagel to the fluid phase transition. In cooling cycle, changes in the slope at ~311 K and ~299 K correspond to the fluid to the gel and the gel to the coagel phase transitions respectively. Errors are smaller than the size of the symbols. (**b**) Variation of <*u*^2^> for DODAB bilayer obtained from elastic intensity scan in both heating and cooling cycles.

FEWS for DODAB vesicles were carried out in the temperature range 285–345 K, in both heating and cooling cycles. *Q*-averaged elastic intensity as a function of temperature is shown in Fig. 3(a). The sharp fall in elastic intensity at 327 K corresponds to the coagel-to-fluid phase transition. In the cooling cycle no sudden jump or change in slope in elastic intensity is observed up to 315 K. Upon cooling further, the slope change around 311 K is attributed to the fluid-to-gel phase transition. Another change in slope is observed at ~299 K, corresponding to the gel-to-coagel phase transition. In the QENS measurements described below, we chose the temperature of 315 K to study the dynamics of the DODAB lipid bilayer in both the coagel and fluid phases (Fig. 3(a)), during heating and cooling cycles respectively. This offers the opportunity to investigate changes in the dynamics of the system solely due to the different phases by ruling out temperature effects.

The dynamical changes through the phase transition can be characterized by monitoring the evolution of the mean-squared displacements (MSD) with temperature. Average MSD, <*u*^2^>, of hydrogen atoms can be obtained from the *Q*-dependence of the elastic intensity, *I*_*el*_(*Q*, *T*) assuming the Gaussian approximation^56^, which is valid mainly at the low *Q* values,

1 $$\mathrm{ln}[\frac{{I}_{el}(Q,T)}{{I}_{el}(Q,{T}_{\min })}]=-\frac{\langle {u}^{2}\rangle {Q}^{2}}{6}$$

The *Q*-dependent elastic scans at the various temperatures are normalized to the elastic scan at the lowest temperature (10 K). Thereafter, the values of <*u*^2^> at each temperature are extracted by fitting with eq. (1) in the *Q*-range 0.5–0.95 Å^−1^. The temperature evolution of the MSD as shown in Fig. 3(b) clearly indicates that the degree of disorder is least in the coagel phase and it is maximum in the fluid phase while it is intermediate in the gel phase^24^.

To get more detailed insights into the involved dynamical processes, QENS experiments were carried out on DODAB vesicles at 315 and 345 K in the heating cycle, where DODAB bilayers are in the coagel and fluid phases respectively, as indicated by elastic intensity scan and DSC measurements (Figs 2 and 3). Besides, QENS measurements were also carried out in the cooling cycles at 330 and 315 K (fluid phase) and at 308 K (gel phase). As explained above, the two QENS measurements at 315 K, namely during heating and cooling, allow to compare the system dynamics in the fluid (during cooling) and coagel (during heating) phases at the same temperature.

**Figure 4:**
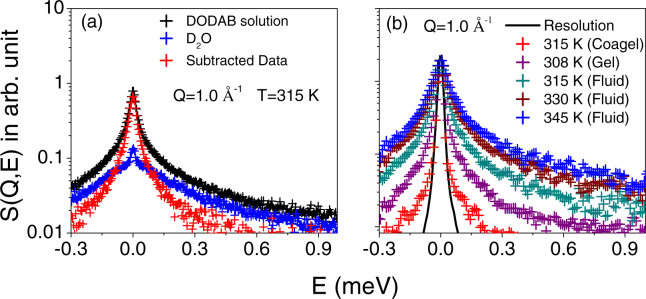
(**a**) QENS spectra for 70 mM DODAB solution and D_2_O at 315 K at *Q* = 1.0 Å^−1^ are shown. The contribution from pure DODAB bilayer after D_2_O subtraction is also shown. (**b**) D_2_O subtracted DODAB spectra are shown at different temperatures at *Q* = 1.0 Å^−1^. Each spectrum is normalized to its peak amplitude. The instrumental resolution is shown by a solid line.

Given the much smaller neutron scattering cross section of deuterium with respect to hydrogen, D_2_O was used as a solvent to minimize its contribution to the QENS data and thereby enhance the contribution from the bilayer. In order to proceed with the data analysis, the D_2_O QENS spectra were subtracted from those of the DODAB solution and the final scattered intensity (*I*_*bl*_) for the DODAB bilayer is obtained using the following relation,

2 $${I}_{bl}(Q,E)={I}_{Solution}(Q,E)-\phi {I}_{{D}_{2}O}(Q,E)$$

where ϕ is the volume fraction of D_2_O. The factor ϕ accounts for the fact that the amount of D_2_O in the DODAB solution is less than that in the pure D_2_O sample. Figure 4(a) shows the quasielastic spectra of DODAB solution, pure solvent (D_2_O) at 315 K and also the D_2_O-subtracted data at a typical *Q* value of 1.0 Å^−1^. Figure 4(b) shows the D_2_O-subtracted spectra of the DODAB bilayer in the coagel phase at 315 K (during heating), in the fluid phase at 345, 330 and 315 K (during cooling), and in the gel phase at 308 K, at a typical *Q* value of 1.0 Å^−1^. The instrumental resolution, as measured using a standard vanadium sample, is also shown in Fig. 4(b). For a direct comparison of the quasielastic broadening, QENS spectra at different temperatures/phases are normalized to the peak intensity of the instrumental resolution. It is clear from Fig. 4(b) that in all the phases, significant quasielastic broadening is observed over the instrumental resolution, suggesting the presence of dynamical motions associated with the DODAB. It is evident that the fluid phase at 315 K shows a much larger broadening than the coagel phase at the same temperature, indicating that motions are quite constrained in the latter phase.

In a neutron scattering experiment, we consider a monochromatic beam of neutrons with energy *E*_*i*_ and wavector ***k***_*i*_ impinging onto a sample. Scattered neutrons are analysed as a function of their final energy *E*_*f*_ and wavevector ***k***_*f*_. Energy transfer and the momentum transfer by the sample can be defined as *E* = *E*_*i*_ − *E*_*f*_ and $\hslash {\bf{Q}}=\hslash {{\bf{k}}}_{i}-\hslash {{\bf{k}}}_{f}$ respectively. In a neutron scattering experiment, information on the molecular motions is obtained by measuring the dynamical structure factor, *S*(*Q*, *E*) which indicates the probability that an incident neutron undergoes a scattering process with energy transfer, *E* and momentum transfer, $\hslash Q$. Incoherent part of the *S*(*Q*, *E*) defined as *S*_*inc*_(*Q*, *E*) deals with the motions of individual atoms. In fact, *S*_*inc*_(*Q*, *E*) is the space and time Fourier transform of the self-correlation function, *G*_*s*_(***r***, *t*)^32^. For a hydrogenous system scattering is mainly incoherent as the incoherent scattering cross section of hydrogen atom is much higher than the coherent or incoherent scattering cross section of any other atom. Since hydrogen constitutes a major component in the DODAB bilayer, we shall consider only the incoherent part of the *S(Q*, *E)*, not mentioning this explicitly hereafter. In the DODAB bilayer, two kinds of motion are expected to contribute within the accessible time scales of the IRIS spectrometer, namely lateral diffusion of the whole DODAB molecule and internal motion within the DODAB molecule. Assuming that these dynamical processes are independent, the scattering law for bilayers can be written as

3 $${S}_{bl}(Q,E)={S}_{lat}(Q,E)\otimes {S}_{\mathrm{int}}(Q,E)$$

where, *S*_*lat*_(*Q*, *E*) and *S*_*int*_(*Q*, *E*) are the scattering functions corresponding to lateral and internal motions of DODAB molecules respectively. The lateral motion of lipids is expected to be characterized by continuous diffusion. The dynamical structure factor for this type of motion is described by a Lorentzian function^30,33,40,43,44^

4 $${S}_{lat}(Q,E)={L}_{lat}({{\rm{\Gamma }}}_{lat},E)$$

where Γ_*lat*_ is the half width at half maximum (HWHM) of the Lorentzian function.

The internal motion of alkyl chains in lipids is instead localized in nature, so there exists a finite probability of finding the hydrogen atoms within the volume of the alkyl chain even after a sufficiently long time. This in turn gives rise to an elastic contribution in the scattering law. Thus, the scattering law for internal motions can be written as,

5 $${S}_{\mathrm{int}}(Q,E)=A(Q)\delta (E)+(1-A(Q)){L}_{\mathrm{int}}({{\rm{\Gamma }}}_{\mathrm{int}},E)$$

The first term in the above equation represents the elastic part. The second term is the quasielastic component which was approximated as a single Lorentzian function, *L*_*int*_(Γ_*int*_, *E*) with half-width at half-maximum (HWHM), Γ_*int*_, that is inversely proportional to the time scale of motion *τ*. The contribution of the elastic scattering out of the total scattering is called the elastic incoherent structure factor (EISF). Therefore, *A(Q)* in Eq. 5 is nothing but the EISF, which represents the space Fourier transform of the particle distribution, taken at infinite time and averaged over all the possible initial positions. The EISF provides information about the geometry of the molecular motion.

**Figure 5:**
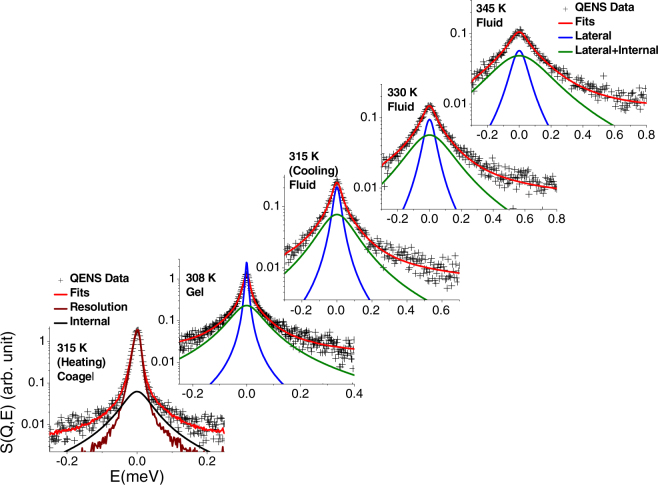
Typical fitted QENS spectra of DODAB bilayers in the coagel phase at 315 K (heating), in the fluid phase at 345, 330, 315 K (cooling) and in the gel phase at 308 K, at *Q* value of 1.41 Å^−1^. The instrumental resolution is shown by a solid line in the lowest panel. Other solid lines correspond to the theoretical descriptions (see text).

The scattering law for DODAB bilayers (Eq. 3) can be written as,

6 $$\begin{array}{rcl}{S}_{bl}(Q,E) & = & {L}_{lat}({{\rm{\Gamma }}}_{lat},E)\otimes [A(Q)\delta (E)+(1-A(Q)){L}_{\mathrm{int}}({{\rm{\Gamma }}}_{\mathrm{int}},E)]\\  & = & A(Q){L}_{lat}({{\rm{\Gamma }}}_{lat},E)+(1-A(Q){L}_{tot}({{\rm{\Gamma }}}_{lat+\mathrm{int}},E)\end{array}$$

where *L*_*lat*_ (Γ_*lat*_, *E*) corresponds to the lateral motion, and *L*_*tot*_ (Γ_*lat+int*_, *E*) corresponds to the combined lateral and internal motions of DODAB, whose HWHM is Γ_*lat+int*_ = Γ_*lat*_ + Γ_*int*_. It is found that Eq. 6 could actually describe the DODAB bilayer spectra quite well, in both gel and fluid phases at all measured *Q* values (Fig. 5). This suggests that both lateral and internal motions of the DODAB lipids are present in the gel and the fluid phases. However, for the coagel phase, Eq. 5 is sufficient to describe the data quite well (Fig. 5 lowest panel), indicating that only internal motion of lipid is active. This is consistent with the fact that in the coagel phase lipid molecules are less hydrated and more densely packed (Fig. 1), resulting in too slow a lateral motion to be observed in the time scale of the IRIS spectrometer. Typical fitted spectra of DODAB vesicles in all the phases, at all the measured temperatures at a *Q* value of 1.41 Å^−1^ are shown in Fig. 5. No *a priori* model for the *Q* dependence is introduced into the fit, neither for the weight factors, nor for the HWHM of the Lorentzian functions. Therefore, the *Q* behaviour of the fit parameters can be used to check the validity of the chosen theoretical models. For the gel and fluid phases, the HWHM corresponding to internal dynamics Γ_*int*_ is obtained by subtracting Γ_*lat*_ from Γ_*lat+int*_.

### Lateral Motion

**Figure 6:**
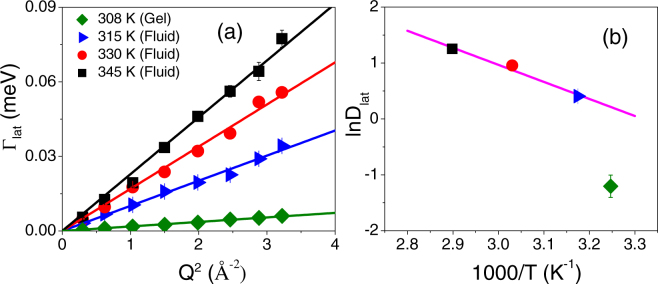
(**a**) Variation of HWHM of Lorentzian corresponding to lateral motion, Γ_*lat*_ with *Q*^2^ at 308 K in the gel and 315, 330 and 345 K in the fluid phase. The solid lines correspond to the Fickian description. (**b**) Plot of lateral diffusion coefficient *D*_*lat*_ for DODAB with inverse temperature. The solid line is the one as per the Arrhenius law in the fluid phase.

As mentioned above, the lateral motion of DODAB lipids is observed in both the gel and fluid phases. The *Q* dependence of Γ_*lat*_ in the fluid phase at 315, 330 and 345 K, and in the gel phase at 308 K is shown in Fig. 6(a). It is evident that in both phases Γ_*lat*_ varies linearly with *Q*^2^, as expected for a continuous diffusion obeying Fick’s law,

7 $${{\rm{\Gamma }}}_{lat}(Q)={D}_{lat}{Q}^{2}$$

where *D*_*lat*_ is the lateral diffusion coefficient of the DODAB molecule. Solid lines in Fig. 6(a) are least squares fits to the experimental points assuming Fick’s law (eq. (7)). The variation of *D*_*lat*_ with temperature, as obtained from the fits, is shown in Fig. 6(b). In the fluid phase, at 315 K, the lateral diffusion coefficient is found to be 1.5 (±0.1) × 10^−6^ cm^2^/s, which increases up to 3.5 (±0.2) × 10^−6^ cm^2^/s at 345 K. In the fluid phase (345–315 K), the lateral diffusion coefficient follows an Arrhenius law, with an activation energy of 6.12 Kcal/mol. In the gel phase, *D*_*lat*_ is found to be 0.3 (±0.1) × 10^−6^ cm^2^/s at 308 K, which is almost an order of magnitude slower than the fluid phase. Indeed in the gel phase, alkyl chains are known to be more ordered and more densely packed than in the fluid phase, where the area per lipid is higher.^57^

### Internal Motion

**Figure 7:**
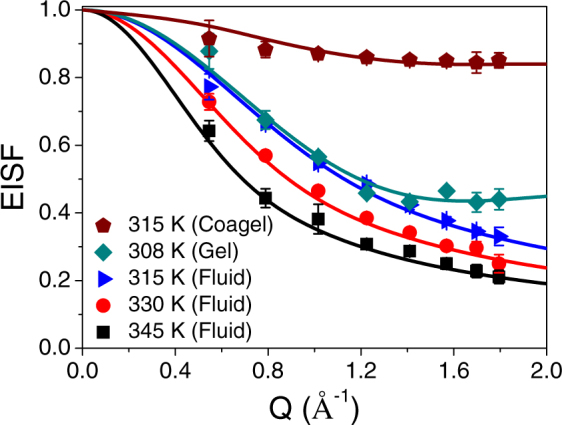
EISF of DODAB bilayer in the coagel, gel and fluid phases. The solid lines represent the least squares fits assuming the models described in the text.

**Figure 8:**
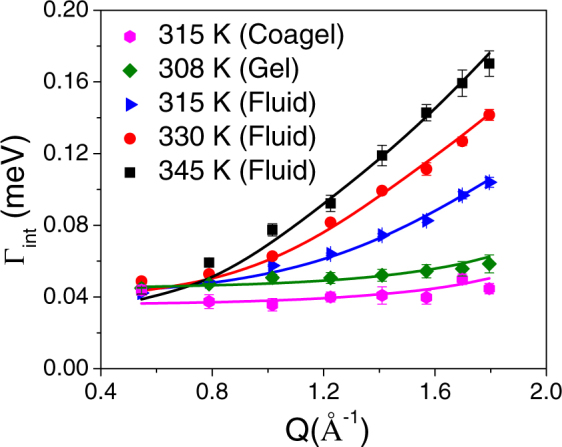
Variation of HWHM, Γ_int_(*Q*), with *Q*, for coagel, gel and fluid phases. The solid lines represent the fitted curves with the models described in the text.

In the coagel phase, only internal motion is observed. However, for gel and fluid phases, both lateral and internal motions are observed. The *Q* dependence of the EISF, *A*(*Q*), and the HWHM, Γ_*int*_(*Q*), corresponding to the internal motion, are shown in Figs 7 and 8 respectively for all the relevant phases. From Fig. 7, it is clear that the EISF is different in the different phases. While the EISF is high it means the system is more ordered, here the coagel phase has the highest EISF, suggesting it to be the most ordered phase. The gel and fluid phases differ particularly in the high-*Q* region. To describe the observed EISF it is required to work out plausible models. A DODAB molecule consists of two methyl units in the head group and two octadecyl alkyl chains (C_18_H_37_). Thus, internal motions of lipids should consist of the motion of methyl units in the head group and the dynamics of the alkyl chains. The resulting scattering law should be a combination of head group motion (*3-fold* reorientation) and motion of the alkyl chain. The scattering law for the motion of hydrogen atoms in methyl groups is well known^33^. As discussed earlier, in both gel and coagel phases, lipid molecules are rather ordered, tightly packed^24–26^, and alkyl chains are in the all-*trans* conformation. In this state, the alkyl chains are expected to perform uniaxial rotational diffusion along their axes. In the corresponding theoretical model, alkyl chain hydrogen atoms undergo reorientation on a circle with a radius of gyration *a*. In absence of any suitable analytical expression of scattering law for powder samples related to uniaxial rotation, one can use the scattering law for jump rotation among *N*_*s*_ equivalent sites, with large *N*_*s*_ (>6) and *Qa* ≤ π^58^. For a powder sample where the *N*_*s*_ equivalent sites are equally distributed on a circle of radius *a*, the incoherent scattering law can be written as^58^,

8 $${S}_{\mathrm{int}}^{uni}(Q,E)={B}_{0}(Qa)\delta (E)+\frac{1}{\pi }\sum _{n=1}^{{N}_{s}-1}{B}_{n}(Qa)\frac{{\tau }_{n}}{1+{E}^{2}{\tau }_{n}^{2}}$$

with elastic structure factor, *B*_0_(*Qa*) and quasielastic structure factor, *B*_*n*_(*Qa*) are,

$$\begin{array}{c}{B}_{0}(Qa)=\frac{1}{{N}_{s}}\sum _{i=0}^{{N}_{s}}{j}_{0}(2Qa\,\sin \,\frac{\pi i}{{N}_{s}})\\ {B}_{n}(Qa)=\frac{1}{{N}_{s}}\sum _{i=1}^{{N}_{s}}{j}_{0}(2Qa\,\sin \,\frac{\pi i}{{N}_{s}})\cos \,\frac{2\pi ni}{{N}_{s}}\end{array}$$

and ${\tau }_{n}^{-1}=2{\tau }^{-1}si{n}^{2}(\frac{n\pi }{{N}_{s}})$. Here *j*_0_ is spherical Bessel function of the zeroth order and *τ* is the average time spent on a site between two successive jumps. In this case, the rotational diffusion constant *D*_*r*_ can be written as,

$${D}_{r}=\frac{2}{\tau }{\sin }^{2}(\frac{\pi }{{N}_{s}})$$

Considering that not all hydrogen atoms in the alkyl chain might be mobile within the observation time scale of the spectrometer at a given temperature, and thus only a fraction *p*_*x*_ of them takes part in the dynamics. The generalized scattering law for internal motion, considering head group and alkyl chain motions for DODAB bilayers in the gel and coagel phases can be written as,

9 $$\begin{array}{c}{S}_{{\rm{int}}}^{gel/coagel}(Q,E)={A}_{gel/coagel}(Q)\delta (E)+\frac{1}{\pi }[\frac{2{P}_{h}}{3}[1-{j}_{0}(Qb)]\frac{3{\tau }_{MG}}{9+{\tau }_{MG}^{2}{E}^{2}}\\ \quad \quad \quad \quad \quad \quad \quad +\,{p}_{x}{P}_{t}\sum _{n=1}^{{N}_{s}-1}{B}_{n}(Qa)\frac{{\tau }_{n}}{1+{\tau }_{n}^{2}{E}^{2}}]\end{array}$$

where *A*_gel/coagel_ (*Q*) is the EISF for the coagel and gel phases and can be written as,

10 $${A}_{gel/coagel}(Q)=\frac{{P}_{h}}{3}(1+2{j}_{0}(Qb))+{P}_{t}(1-{p}_{x})+{P}_{t}[\frac{{p}_{x}}{{N}_{s}}\sum _{i=1}^{{N}_{s}}{j}_{0}(2Qa\,\sin \,\frac{\pi i}{{N}_{s}})]$$

*P*_*h*_ is the fraction of hydrogen atoms in head group and *P*_*t*_ is the fraction of hydrogen atoms in alkyl chain. For the DODAB molecule ((C_18_H_37_)_2_N^+^(CH_3_)_2_Br^−^), *P*_*h*_ and *P*_*t*_ are equal to 6/80 and 74/80 respectively. Here *b* is the H-H distance (1.8 Å) in the methyl group and *τ*_*MG*_ is the mean residence time of a hydrogen atom in a methyl group. Here, we have taken *N*_*s*_ = 12, so that the number of sites is sufficiently large for mimicking a uniaxial rotational diffusion model in the given *Q* range^58^.

A least-squares fitting method was employed to describe the experimental EISF of the coagel and gel phases, using eq. (10) with *p*_*x*_ and *a* as fit parameters. The resulting fitting curves, shown in Fig. 7, are in very good agreement with the experimental data. In the coagel phase, *p*_*x*_ and *a* are found to be 0.15 and 1.7 ± 0.1 Å respectively. This indicates that about 15% of the hydrogen atoms in the alkyl chains of DODAB molecule undergo uniaxial rotation with a radius of gyration of 1.7 Å, which is actually close to the average distance of hydrogen atoms from the chain axis. In the gel phase, *p*_*x*_ and *a* are found to be 0.64 and 1.8 ± 0.1 Å respectively. Therefore the fraction of mobile hydrogen atoms increases sharply from 15% to 64% in the gel phase compared to the coagel phase, which is in agreement with the fact that packing density of alkyl chains decreases on going from coagel to gel phase^24^. In particular it was found that the radius of gyration is similar in both the phases, thus confirming the robustness of the chosen model. Also, the proportion of mobile fraction as obtained from the QENS analysis is quite consistent with the MSD values obtained from the FEWS in the gel and coagel phases (Fig. 3(b)). The fact that the uniaxial rotational diffusion model could describe the internal motion in the coagel and gel phases and the correlation of the packing density of alkyl chains with the fraction of mobile hydrogen indicate that the dynamical model described here is consistent with the structure.

The rotational diffusion coefficient *D*_*r*_ and τ_MG_ can be obtained for both phases by a least-squares fitting of eq. (9) to the experimental HWHM. The obtained values for *D*_*r*_ are 5.3 (±0.2) × 10^10^ s^−1^ and 6.8 (±0.3) × 10^10^ s^−1^, in the coagel and gel phase respectively. Mean residence times for hydrogen in methyl group, τ_*MG*_ are found to be 6.7 ± 0.3 ps and 5.2 ± 0.2 ps in the coagel and gel phases respectively. In the coagel phase, the slower rotational diffusion for alkyl chain and higher residence time of hydrogen in head group can again be ascribed to the lower hydration level and the denser packing of the lipid molecules with respect to the gel phase^24,26^.

In the fluid phase, DODAB molecules are more disordered and loosely packed^24^ with high degree of *gauche* defects along the alkyl chain compared to the other phases. As to the alkyl chains in the fluid phase, their dynamics could involve various kinds of motion, including chain reorientation, conformational changes, bending modes, stretching modes and so forth. The superposition of all these motions can be effectively modeled by assuming that the hydrogen atoms of each CH_2_ unit undergo localized translational diffusion (LTD) confined within spherical domains. In fact, we have already employed the LTD model to successfully describe the dynamics of alkyl chains in various molecular aggregates, such as micelles, vesicles and lipid membranes^35–41,43,44,47^. The observed *Q* dependence of the EISF and of Γ_*int*_, as shown in Figs 7 and 8, is also indicative of the LTD model. Indeed, Γ_*int*_ increases with increasing *Q* and flattens towards a finite nonzero value in the zero-*Q* limit, which is a typical signature of the LTD model. Due to flexibility of the alkyl chains, the hydrogen atoms may not all have the same diffusivities and same domain sizes. Therefore we assume a simple linear distribution of radii and diffusivities The CH_2_ unit nearest to the head group diffuses in a sphere of smallest radius and lowest diffusivity. As one progresses towards the tail of the alkyl chain, radius and diffusivity increase. The largest radius and diffusivity are associated with the very end of the chain. The scattering law for particles diffusing within a sphere is given by Volino and Dianoux^59^. The generalized scattering law for the dynamics of alkyl chains assuming linearly distributed radii and diffusivities can be written as^33,36,59^,

11 $$\begin{array}{rcl}{S}_{\mathrm{int}}^{LTD}(Q,E) & = & \frac{1}{{N}_{c}}\sum _{i=1}^{{N}_{c}}[{A}_{0}^{0}(Q{R}_{i})\delta (E)\\  &  & +\,\frac{1}{\pi }\sum _{\{l,n\}\ne \{0,0\}}(2l+1){A}_{n}^{l}(Q{R}_{i})\frac{({x}_{n}^{l}){D}_{i}/{R}_{i}^{2}}{{[({x}_{n}^{l}){D}_{i}/{R}_{i}^{2}]}^{2}+{E}^{2}}]\end{array}$$

The first term corresponds to the elastic component, while the second term accounts for the quasielastic contribution, which comprises a series of Lorentzian functions. ${A}_{0}^{0}(QRi)={[\frac{3{j}_{1}(Q{R}_{i})}{Q{R}_{i}}]}^{2}$ and ${A}_{n}^{l}$(*QR*_*i*_) (*n*, *l* ≠ 0, 0) are the elastic and quasielastic structure factors respectively. ${A}_{n}^{l}$(*QR*_*i*_) for different *n* and *l* can be calculated by using the values of *x*_n_^*l*^ listed in ref.^59^. *R*_*i*_ and *D*_*i*_ are the radius of the sphere and the diffusivity associated with the *i*^*th*^ site of the alkyl chain, with *i* increasing from the head towards the tail, and can be written as:

$${R}_{i}=\frac{i-1}{{N}_{c}-1}[{R}_{\max }-{R}_{\min }]+{R}_{\min }$$

and

$${D}_{i}=\frac{i-1}{{N}_{c}-1}[{D}_{\max }-{D}_{\min }]+{D}_{\min }$$

where *N*_c_ = 18 is the total number of CH_2_ units in the alkyl chain. Therefore, the total scattering law for the internal dynamics of the DODAB molecules in fluid phase is taken as a combination of *3-fold* rotation of the methyl head groups and localized translational diffusion of the alkyl chain (eq. (11)). The generalized scattering law, considering various components of motion with appropriate weight factors, for the internal motion of DODAB bilayers in the fluid phase can be written as:

12 $$\begin{array}{rcl}{S}_{\mathrm{int}}^{fluid}(Q,E) & = & \{\frac{{P}_{h}}{3}[1+2{j}_{0}(Qb)]+\frac{{P}_{t}}{{N}_{c}}\sum _{i=1}^{{N}_{c}}{A}_{0}^{0}(Q{R}_{i})\}\delta (E)\\  &  & +\,\frac{1}{\pi }[\frac{2{P}_{h}}{3}[1-{j}_{0}(Qb)]\frac{3{\tau }_{MG}}{9+{\tau }_{MG}^{2}{E}^{2}}\\  &  & +\,\frac{{P}_{t}}{{N}_{c}}\sum _{i=1}^{{N}_{c}}\sum _{\{l,n\}\ne \{0,0\}}\{(2l+1){A}_{n}^{l}(Q{R}_{i})\frac{({x}_{n}^{l}){D}_{i}/{R}_{i}^{2}}{{[({x}_{n}^{l}){D}_{i}/{R}_{i}^{2}]}^{2}+{E}^{2}}\}\end{array}$$

For DODAB (with P_h_ = 6/80, P_t_ = 74/80 and N_c_ = 18), the resultant EISF for the fluid phase can be written as,

13 $${A}_{fluid}(Q)=\frac{1}{80}\{\frac{6}{3}[1+2{j}_{0}(Qb)]+\frac{74}{18}\sum _{i=1}^{18}{[\frac{3{j}_{1}(Q{R}_{i})}{Q{R}_{i}}]}^{2}\}$$

where the radii *R*_*i*_, including the smallest *R*_*min*_ (in the head group) and the largest *R*_*max*_ (at the tail end), are determined by a least squares fitting of eq. (13) to the experimental EISF. As evident from the solid lines in Fig. 7, the above model describes the observed EISF quite well in the fluid phase. The values of *R*_*min*_ are found to be unrealistically small, which essentially reflect the very small movement of the hydrogen atoms in the CH_2_ units close to the head group. Values of *R*_*max*_ at different temperatures are given in Table 1. At 315 K, *R*_*max*_ is found to be 3.3 (±0.3) Å. With increasing temperature, the value of *R*_*max*_ also increases and reaches up to 5.4 (±0.3) Å at 345 K.

**Table 1:** Dynamical parameters for internal motion as obtained from the fit of the QENS data at different temperatures in the fluid phase of DODAB bilayer.

Temperature	*D*_*int*_ × 10^−6^ (cm^2^/s)		*R*_*max*_	*τ*_*MG*_
(K)	*D*_*min*_	*D*_*max*_	(Å)	*(ps)*
315	0.16 (±0.3)	12.8 (±0.5)	3.3 (±0.2)	4.4 (±0.2)
330	0. 6 (±0.3)	19.4 (±0.5)	4.2 (±0.2)	3.2 (±0.2)
345	0.6 (±0.3)	26.1 (±0.5)	5.4 (±0.3)	2.6 (±0.1)

To estimate the time scale of DODAB internal motions, we carried out a detailed analysis of the HWHM *Q* dependence (Γ_*int*_ (*Q*)). Since no analytical expression exists for Γ_*int*_ (*Q*), it can be numerically calculated by means of eq. (12), for given values of *R*_min_, *R*_max_, *D*_min_, *D*_max_ and *τ*_MG_. *R*_min_ and *R*_max_ are obtained from the previous analysis of the EISF *Q* dependence and used as such for the calculation of HWHM. A least-squares method is then used to fit the observed Γ_int_ with *D*_min_, *D*_max_ and *τ*_MG_ as free parameters. As shown by the solid lines in Fig. 8, the model describes quite well the experimental HWHM. The obtained best-fit parameters are listed in Table 1.

The results of this study can be compared with existing studies on the dynamics of the other model membrane systems^33,34,60–62^. One of the first QENS studies on a supported dipalmitoylphosphatidylcholine (DPPC) lipid bilayer by Pffeifer *et al*.^60^ proposed a description of lipid dynamics based on lateral, rotational and librational motions of the molecule. It was widely accepted that the lateral motion of lipids followed the continuous diffusion model. However, Busch *et al*.^34^ proposed a ballistic flow-like motion to explain the overall long-range diffusion of phospholipid molecules. But the recent studies by Rheinstadter^61^ group and Sharma *et al*.^33^ on dimyristoylphosphatidylcholine (DMPC), have shown that the lateral motion of lipids in length scales larger than lipid diameter is found to be well described by continuous diffusion. Here, we have also found that the lateral motion of DODAB lipid follows the same model. Sharma *et al*.^33^ have also shown that the lateral diffusivity of the DMPC is an order of magnitude higher in the fluid phase (0.77 × 10^−6^ cm^2^/s) compared to the gel phase (0.07 × 10^−6^ cm^2^/s). The diffusion coefficients for lateral motion of the DODAB lipids obtained in this study effectively show similar trend in transition from fluid to the gel phase. QENS study on DMPC and 1-palmitoyl-oleoyl-sn-glycero-phosphocholine (POPC) in the fluid phase has also been carried out by Wanderlingh *et al*.^62^. The values of lateral diffusion coefficient for DMPC and POPC lipid are reported to be 1.2 × 10^−6^ cm^2^/s and 1.9 × 10^−6^ cm^2^/s respectively. It is to be noted that the lipid dynamics depend on various parameters such as physical state of the membrane, concentration, temperature, hydration level, etc. In the present study the lateral diffusion coefficients of DODAB in the gel and fluid (315 K) phases are found to be 0.3 × 10^−6^ cm^2^/s and 1.5 × 10^−6^ cm^2^/s respectively. Comparing the internal motion of different model membrane systems, the diffusion coefficient corresponding to internal dynamics in the fluid phase of DODAB and DMPC lipid are found to be 13 × 10^−6^ cm^2^/s and 7.9 × 10^−6^ cm^2^/s^33^ respectively.

### Molecular Dynamics (MD) Simulation

MD simulations allow computing the atomistic dynamics of condensed systems on the same spatial and temporal domains probed by QENS experiments. As such, they provide an excellent tool to validate theoretical models employed for QENS data analysis. To this end, we carried out MD simulations of a DODAB bilayer at two temperatures, namely 298 and 350 K, corresponding to the ordered and fluid phases respectively. From simulations carried out with different initial conditions, it is observed that the results are independent of the initial conditions.

**Figure 9:**
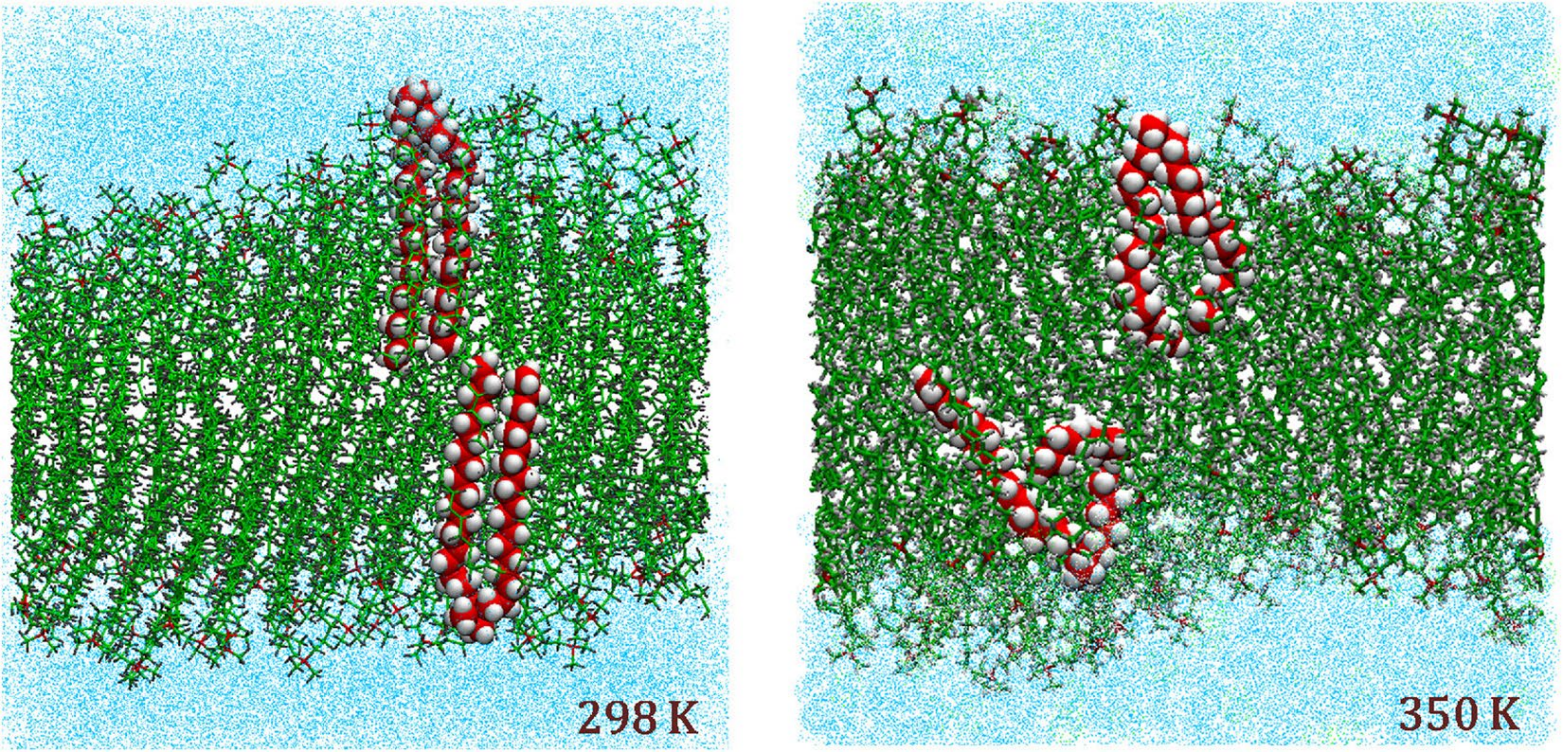
Snapshots of simulated DODAB bilayer at 298 K and 350 K after an equilibration of 15 ns. Conformations of two randomly selected DODAB molecules in each phase are highlighted.

**Figure 10:**
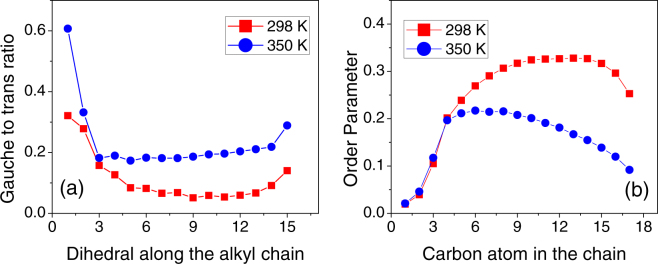
(**a**) G*auche* to *trans* ratio of a DODAB lipid molecule, (**b**) Order parameter as defined in equation (14) at 298 K (ordered phase) and at 350 K (fluid phase).

At 298 K, it was observed that the monomers of the DODAB bilayer remained in almost all *trans* configuration (Fig. 9), which is consistent with the results of an earlier MD simulation work by Jamróz *et al*.^20^. On the other hand, at 350 K, a significant increase in *gauche* defects is observed suggesting the onset of chain disorder (Fig. 9). This chain disorder can be quantified by calculating the *gauche* to *trans* ratio along the alkyl chain of the molecule. Each individual dihedral angle (φ) can be classified as *gauche* if φ is in the range 45° to 75° and *trans* if φ falls in the range 165° to 195°. Figure 10(a) shows the ratio of *gauche*-to-*trans* dihedral bonds that characterize the *gauche* defect in the chain. In the low temperature phase, apart from the carbon near the headgroup, the alkyl chain is found in an almost all-*trans* configuration. This phase is reminiscent of an ordered phase found before the main transition in bilayer systems^20^. The higher *gauche* defects near the headgroup is a consequence of the repulsion of CH_2_ units connected to the ammonium headgroup. This is in compliance with earlier simulation results, carried out at 298 K, which find that the molecules in the bilayer takes a open-scissor conformation and show a kink near the headgroup^20^. However, at 350 K, we find that there is a substantial increase in number of *gauche* defects, indicative of a fluid phase found in lipid bilayer systems. The average *gauche*-to-*trans* ratio at 298 K is as low as 0.11, whereas in the fluid phase (350 K) it reaches up to 0.24, meaning that the number of dihedral bonds in *gauche* conformation is more than doubled. Such an increase in *gauche* defects indicates that the DODAB lipids are more loosely packed in the fluid phase.

The alkyl chain order parameter (*S*_*CH*_) is an important quantity used to characterize the transition to the fluid phase in lipid bilayers. In MD simulations, it can be obtained from the trajectory of particles using the equation,

14 $${S}_{CH}^{i}=\frac{1}{2}[3\langle {\cos }^{2}{\theta }_{i}\rangle -1]$$

where *θ*_*i*_ is the angle between the C-H bond vector and a reference axis (here, the bilayer normal) for the *i*^*th*^ carbon atom in the chain, and the average is over all the molecules and time steps of the simulation. Figure 10(b) shows the *S*_*CH*_ parameter calculated from MD simulation trajectories for the two different phases. It is observed that the S_CH_ parameter is almost same in both the phases up to about the 4^th^ carbon atom, which is due to the molecular structure of DODAB. It is found that beyond the 4^th^ carbon the order parameter in the fluid phase is significantly lower compared to the ordered phase (Fig. 10(b)).

Comparison of MD simulation results with QENS data is generally performed by calculating the intermediate scattering function *I*(*Q*, *t*) from the MD simulation trajectories, and then comparing its dynamical features with those of the experimental *S*(*Q*, *E*). The (incoherent) dynamical structure factor *S*(*Q*, *E*) is the Fourier transform of the (incoherent) intermediate scattering function, *I*(*Q*, *t*) and can be written as^32^,

15 $$\begin{array}{c}S(Q,E)=\frac{1}{2\pi }\underset{-{\rm{\infty }}}{\overset{{\rm{\infty }}}{\int }}I(Q,t){e}^{-\frac{iEt}{\hslash }}dt\,\,\\ I(Q,t)=\overline{\langle {e}^{i{\bf{Q}}.({\bf{r}}({t}_{0}+t)-{\bf{r}}({t}_{0}))}\rangle }\,\,\end{array}$$

where ***r***(*t*) is the position vector of the hydrogen atom in DODAB, and ***Q*** is the momentum transfer. The angular brackets indicate an ensemble average and the bar denotes the average over all possible orientations of the ***Q*** vectors. The incoherent intermediate scattering function, *I*(*Q*, *t*), is readily computed from an MD trajectory using above equation. However, calculating the inverse Fourier transform of the experimentally obtained *S*(*Q*, *E*) will introduce spurious errors due to limited and finite energy transfer range of the spectrometer. Therefore a model comparison is rather less error prone. It is known that the time and energy domains have an inverse relationship with each other, therefore the features of *I*(*Q*, *t*) calculated from MD simulation trajectories in the $t\to 0$ limit, is reflected in higher energy transfers in *S*(*Q*, *E*) which correspond to fast relaxation modes of atoms in the system. In the experimentally obtained *S*(*Q*, *E*), this depends on the energy transfer window of the spectrometer being used. On the other hand, the energy resolution of the spectrometer limits the slowest relaxation mode observable in the system, which is observed in the long time behaviour of *I*(*Q*, *t*).

As mentioned above that at 298 K the DODAB bilayers exist in the ordered phase. Whereas at 350 K DODAB bilayer is found to be in the disordered state, which is much above the transition temperature (327 K) as observed in DSC and FEWS (Figs 2 and 3) where the system is said to be in the fluid phase. QENS data recorded at 345 K showed large quasielastic broadening, data analysis revealed two distinct motions occurring in DODAB bilayers on different time scales: lateral motion of lipid molecules and localized internal motion of the atoms within the lipid chain. However, we found that two decaying exponentials (related to two time scales) are insufficient to model the *I*(*Q*, *t*) obtained from the MD simulation of the fluid phase. In view of our recent work on micelles^48^, a model based on three different motions, namely lateral, internal and fast (presumably torsional), was successfully able to account for the MD simulation data,

16 $$I(Q,t)={e}^{-{{\rm{\Gamma }}}_{lat}t}[{{\rm{A}}}_{1}+(1-{{\rm{A}}}_{1}){e}^{-{{\rm{\Gamma }}}_{\mathrm{int}}t}][{{\rm{A}}}_{2}+(1-{{\rm{A}}}_{2}){e}^{-{{\rm{\Gamma }}}_{fast}t}]$$

**Figure 11:**
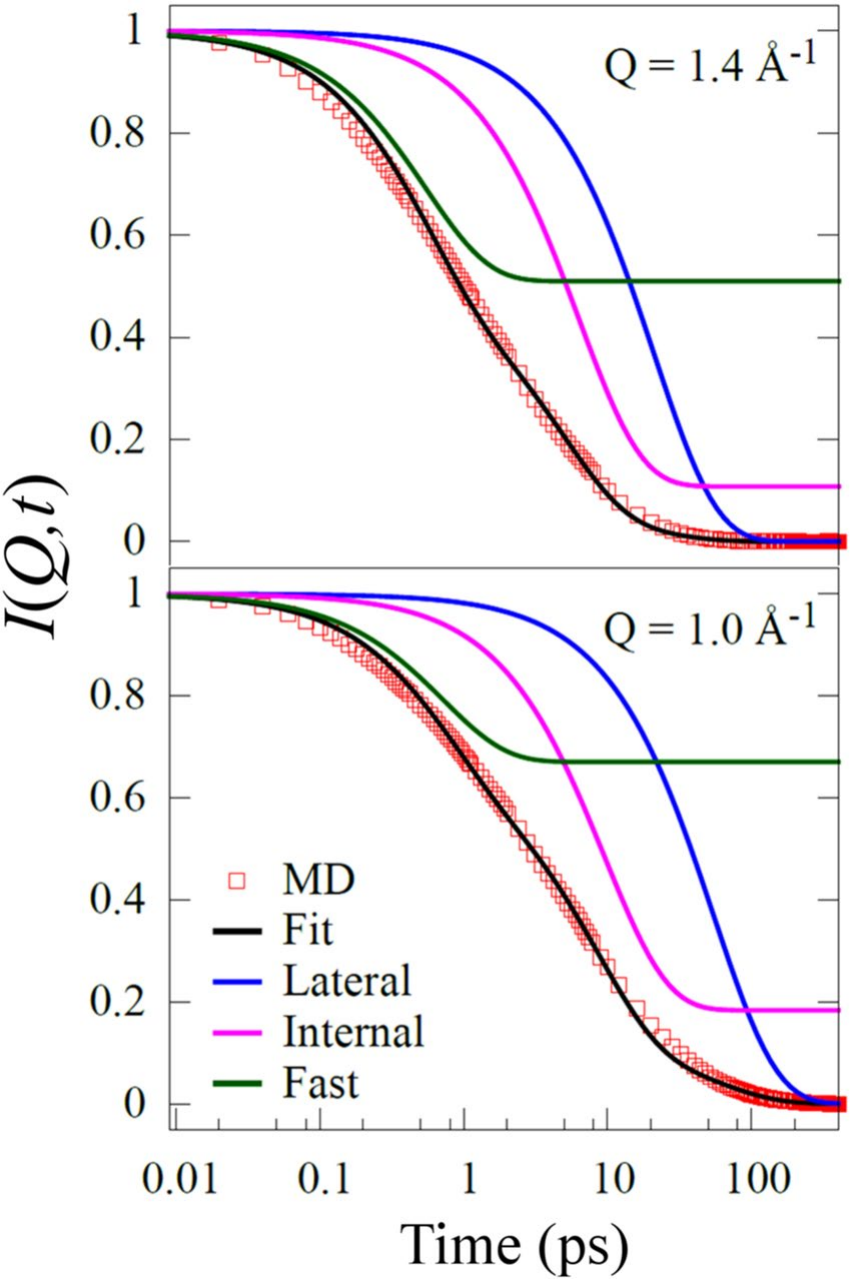
*I*(*Q*, *t*) for DODAB molecules with their fits assuming equation (16) and the individual components at typical two different *Q*-values.

**Figure 12:**
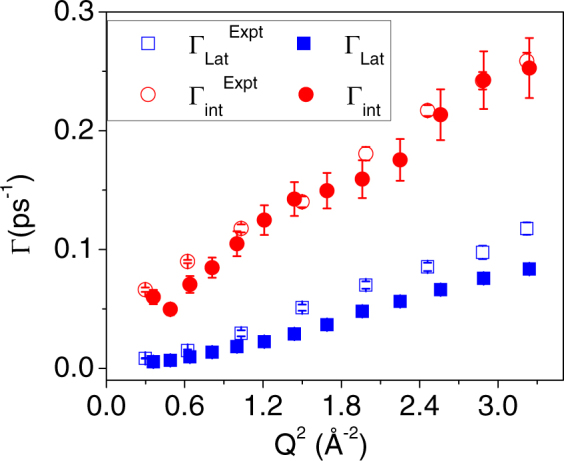
*Q* dependence of ${{\rm{\Gamma }}}_{{lat}}$ and ${{\rm{\Gamma }}}_{{int}}$ with respect to from fits of MD simulation (filled) and QENS experiments (empty).

Here, Γ_*lat*_, Γ_*int*,_ and Γ_*fast*_ are the decay constants associated with lateral, internal and fast motions respectively in the DODAB bilayer system. Typical fits of *I*(*Q*, *t*) at different *Q* values are shown in Fig. 11. As discussed earlier, the fast motion ($\hslash$Γ_*fast*_ ~ 1 meV) observed in MD simulation lies outside the energy transfer window of the IRIS spectrometer and therefore it is not expected to contribute to the experimental data. On the other hand, the time scales corresponding to lateral and internal motions obtained from the simulated *I*(*Q*, *t*) match quite well with those obtained from QENS experimental data. Figure 12 shows the *Q* dependence of Γ_*lat*_ and Γ_*int*_, as obtained from both MD simulations and QENS experiments at 350 and 345 K respectively. The agreement between experiment and simulations is rather impressive. Indeed, the diffusivity calculated from simulated data for the lateral motion of DODAB lipids amounts to *D*_*lat*_ = 3.2 (±0.2) × 10^−5^ cm^2^/s, which is very close to the value derived from the experimental data (3.5 (±0.2) × 10^−5^ cm^2^/s) at 345 K. These results are well supported by the physical model chosen to interpret our QENS data on DODAB bilayers.

### Conclusions

The structure and dynamics of lipid-bilayer systems are of great interest, due to their biomimetic membrane properties and extensive applications in drug delivery and gene/DNA transfection. Here we report a first extensive and detailed study about the microscopic dynamics of DODAB bilayers across their complex phase diagram, encompassing coagel, gel and fluid phases. We derived a comprehensive picture by means of the combined use of a wide range of techniques, namely differential scanning calorimetry, neutron fixed elastic window scan, quasielastic neutron spectroscopy and molecular dynamics simulation.

Neutron fixed elastic window scan and differential scanning calorimetry independently provide a consistent picture for the DODAB phase diagram. Both methods reveal the presence of a hysteresis phenomenon. At low temperature the coagel phase is the stable one. Upon heating, the system passes from the coagel to the fluid phase at 327 K. Upon cooling back, on the other hand, the fluid phase persists down to 311 K, where a fluid-to-gel transition takes place. Only upon further cooling, below about 299 K, the system undergoes a second transition that finally takes the system from the intermediate gel phase back to the coagel phase.

QENS measurements unravel the details of the microscopic dynamics underlying each of the different structural phases by pinpointing their specific dynamical peculiarities. In fact, at the atomic scale a complex dynamical behavior emerges, the interpretation of which requires advanced theoretical models. From the QENS data analysis, two main dynamical contributions emerge: the lateral diffusion of the whole DODAB molecule within the leaflet, and the internal motion of the DODAB lipid chains. In the coagel phase, only the internal modes were detected. The viscosity (or rigidity) of the system is indeed so large in this phase that lateral diffusion is either frozen or too slow to be observable within the time window of the spectrometer (~80 ps). They become visible in the intermediate gel phase, where they follow the typical Fickian behavior of continuous diffusion. At the same time, internal motion is enhanced, as both the fraction *p*_*x*_ of mobile hydrogen atoms available for uniaxial rotation and their rotational diffusion coefficient *D*_*r*_ increase with respect to the coagel phase (*p*_*x*_ grows from 15% to 64%, and *D*_*r*_ from 5.3 × 10^10^ s^−1^ to 6.8 × 10^10^ s^−1^). In the fluid phase, both lateral diffusion and internal motions are strongly boosted. Lateral diffusion becomes about one order of magnitude faster than in the gel phase, with the diffusion coefficient following a smooth Arrhenius temperature dependence and reaching up to 3.5 × 10^−6^ cm^2^/s at 345 K. In the meantime, internal motions become so richly populated that uniaxial rotation alone is not adequate to fully interpret the data. In the fluid phase the dynamics of alkyl chains can indeed involve more complex movements, including chain reorientations, bending modes, stretching modes and even conformational jumps. The superposition of all these motions can be effectively modeled by assuming that each hydrogen atom of the alkyl chain undergoes confined translational diffusion within spherical domains. Due to the high flexibility of alkyl chains in this phase, each atom is characterized by its own diffusion coefficient and diffusion domain. A simplified linear distribution of diffusivities and domain radii, progressively increasing from the head toward the tail of the alkyl chain, is perfectly adequate to fully account for the details of the experimental data.

The results of MD simulation of the DODAB lipid bilayers indicate that at 298 K the alkyl chain of the DODAB molecules in the bilayers are in an almost all-*trans* configuration. However, at 350 K, it is observed that significant *gauche* defects are introduced along the alkyl chain and the system resembles the fluid phase in bilayer systems. The rise in *gauche*-to-*trans* ratio from the low to high temperature phase is connected with a progressively lower lipid packing fraction, a larger area per lipid, therefore with a globally higher level of structural and conformational disorder in the system. The complex dynamical picture of the fluid phase emerging from the experimental data is fully confirmed and explained by the molecular dynamics simulations. The whole of this information strongly supports the chosen physical interpretation that suggested adopting a progressively more complex and mobile dynamical models to describe the QENS data. The chosen dynamical models are well supported by the direct calculation of the intermediate scattering function *I*(*Q*, *t*) from the simulated atomic trajectories where it is characterized by three relaxation times: a fast one, that falls outside the energy window of the QENS experiment, and two slower ones, which perfectly match the experimental time scales of lipid lateral diffusion and internal motion respectively. The quantitative agreement between the experimental diffusion coefficients and those worked out from the computed relaxation times is strikingly good.

In summary, the detailed dynamical and phase behavior of DODAB bilayers presented here demonstrate a thermotropic evolution of the dynamics in this biomimetic membrane. Since the general behavior of biological membranes and in particular the release rate of drugs or genetic material, are strongly affected by the dynamics of lipid monomers, the present study should be of profound interest for all kind of applications involving lipid bilayers.
